# Supplementary material for: Crawling and Gliding: A Computational Model for Shape-Driven Cell Migration
Source: PLoS Comput Biol. 2015 Oct 21;11(10):e1004280. doi: 10.1371/journal.pcbi.1004280 (PMC4619082; doi:10.1371/journal.pcbi.1004280)
Supplement: S1 Code — (ZIP) [file pcbi.1004280.s012.zip › release/tst/doc/html/output_8cpp.html]

Tissue Simulation Toolkit: output.cpp File Reference


Tissue Simulation Toolkit
 0.1.4.1


- Main Page
- Namespaces
- Classes
- Files

- File List
- File Members

Macros |
Functions

output.cpp File Reference

`#include <stdio.h>`  
`#include <string.h>`  
`#include <stdlib.h>`  
`#include <errno.h>`  
`#include <sys/types.h>`  
`#include <sys/stat.h>`  
`#include "warning.h"`  
`#include "parameter.h"`  
`#include "output.h"`

Include dependency graph for output.cpp:

|  |  |
| --- | --- |
| Macros | |
| #define | FNAMESIZE   100 |
|  | |
| #define | INITIAL\_BUFSIZE   100 |
|  | |

|  |  |
| --- | --- |
| Functions | |
| int | OpenFileAndCheckExistance (FILE \*\*fp, const char \*fname, char \*ftype) |
|  | |
| int | FileExistsP (const char \*fname) |
|  | |
| int | YesNoP (const char \*message) |
|  | |
| FILE \* | OpenWriteFile (const char \*filename) |
|  | |
| FILE \* | OpenReadFile (const char \*filename) |
|  | |
| char \* | ReadLine (FILE \*fp) |
|  | |
| void | CheckFile (FILE \*fp) |
|  | |
| char \* | Chext (char \*filename) |
|  | |

## Macro Definition Documentation

#define FNAMESIZE   100

Referenced by OpenWriteFile().

#define INITIAL\_BUFSIZE   100

Referenced by ReadLine().

## Function Documentation

|  |  |  |  |  |  |
| --- | --- | --- | --- | --- | --- |
| void CheckFile | ( | FILE \* | *fp* | ) |  |

References errno, and error().

Referenced by ReadLine().

|  |  |  |  |  |  |
| --- | --- | --- | --- | --- | --- |
| char\* Chext | ( | char \* | *filename* | ) |  |

|  |  |  |  |  |  |
| --- | --- | --- | --- | --- | --- |
| int FileExistsP | ( | const char \* | *fname* | ) |  |

References FALSE, and TRUE.

Referenced by OpenWriteFile().

|  |  |  |  |
| --- | --- | --- | --- |
| int OpenFileAndCheckExistance | ( | FILE \*\* | *fp*, |
|  |  | const char \* | *fname*, |
|  |  | char \* | *ftype* |
|  | ) |  |  |

References FALSE, and TRUE.

Referenced by OpenReadFile().

|  |  |  |  |  |  |
| --- | --- | --- | --- | --- | --- |
| FILE\* OpenReadFile | ( | const char \* | *filename* | ) |  |

References FALSE, and OpenFileAndCheckExistance().

Referenced by Parameter::Read().

|  |  |  |  |  |  |
| --- | --- | --- | --- | --- | --- |
| FILE\* OpenWriteFile | ( | const char \* | *filename* | ) |  |

References FileExistsP(), FNAMESIZE, and TRUE.

|  |  |  |  |  |  |
| --- | --- | --- | --- | --- | --- |
| char\* ReadLine | ( | FILE \* | *fp* | ) |  |

References CheckFile(), errno, error(), INITIAL\_BUFSIZE, and MEMORYCHECK.

Referenced by SearchToken(), and SkipLine().

|  |  |  |  |  |  |
| --- | --- | --- | --- | --- | --- |
| int YesNoP | ( | const char \* | *message* | ) |  |

References FALSE, and TRUE.


---

Generated on Thu Aug 14 2014 22:04:01 for Tissue Simulation Toolkit by  

 1.8.6
